# Suicides Among American Indian or Alaska Native Persons — National Violent Death Reporting System, United States, 2015–2020

**DOI:** 10.15585/mmwr.mm7137a1

**Published:** 2022-09-16

**Authors:** Deborah Stone, Eva Trinh, Hong Zhou, Laura Welder, Pamela End of Horn, Katherine Fowler, Asha Ivey-Stephenson

**Affiliations:** ^1^Division of Injury Prevention, National Center for Injury Prevention and Control, CDC; ^2^Indian Health Service, Rockville, Maryland; ^3^Division of Violence Prevention, National Center for Injury Prevention and Control, CDC.

Compared with the general U.S. population, American Indian or Alaska Native (AI/AN) persons, particularly those who are not Hispanic or Latino (Hispanic) AI/AN, are disproportionately affected by suicide; rates among this group consistently surpass those among all other racial and ethnic groups (*1*). Suicide rates among non-Hispanic AI/AN persons increased nearly 20% from 2015 (20.0 per 100,000) to 2020 (23.9), compared with a <1% increase among the overall U.S. population (13.3 and 13.5, respectively) (*1*). Understanding characteristics of suicide among AI/AN persons is critical to developing and implementing effective prevention strategies. A 2018 report described suicides in 18 states among non-Hispanic AI/AN persons only (*2*). The current study used 2015–2020 National Violent Death Reporting System (NVDRS) data among 49 states, Puerto Rico, and the District of Columbia to examine differences in suicide characteristics and contributing circumstances among Hispanic and non-Hispanic AI/AN populations, including multiracial AI/AN. Results indicated higher odds across a range of circumstances, including 10 of 14 relationship problems (adjusted odds ratio [aOR] range = 1.2–3.8; 95% CI range = 1.0–5.3) and six of seven substance use problems (aOR range = 1.2–2.3; 95% CI range = 1.1–2.5), compared with non-AI/AN persons. Conversely, AI/AN decedents had reduced odds of having any current known mental health condition, any history of mental health or substance use treatment, and other common risk factors (aOR range = 0.6–0.8; 95% CI = 0.2–0.9). Suicide is preventable. Communities can implement a comprehensive public health approach to suicide prevention that addresses long-standing inequities affecting AI/AN populations (*3*).

NVDRS is a state-based surveillance system that collects information from death certificates, coroner or medical examiner reports, and law enforcement reports on the characteristics and circumstances of violent deaths, including suicides (*4*). Data in this report are from the District of Columbia, Puerto Rico, and 49 U.S. states participating in NVDRS during 2015–2020*; some jurisdictions did not participate for the entire period because they were not yet funded or because they did not achieve data completion thresholds (Supplementary Table, https://stacks.cdc.gov/view/cdc/121071) (*4*). Analyses were limited to decedents aged ≥10 years, because determining suicide intent in young children can be difficult (*5*). AI/AN persons are defined in NVDRS as persons with origins among any of the original peoples of North America and who maintain cultural identification through tribal affiliation or community recognition (Alaska Natives are included among this group) (*6*). For this study, characteristics and circumstances of suicide were compared among decedents with any AI/AN identification, similar to a recent analysis of homicides among AI/AN persons (*7*). Rural-urban commuting area codes were used to determine nonmetropolitan and metropolitan geographic areas. All comparisons between AI/AN and non-AI/AN persons were examined using Pearson’s chi-square tests (with p<0.05 considered statistically significant) and logistic regression analyses, controlling for age and sex to estimate aORs with 95% CIs. Analyses were conducted using SAS (version 9.4; SAS Institute). This analysis was reviewed by CDC and was conducted consistent with applicable federal law and CDC policy.^†^

During 2015–2020, a total of 3,397 suicides among AI/AN persons and 179,850 suicides among non-AI/AN persons were recorded in NVDRS (Table 1). Approximately three quarters (74.6%) of AI/AN suicide decedents were aged ≤44 years, compared with less than one half (46.5%) of non-AI/AN decedents. The highest percentage of AI/AN suicides (46.9%) occurred among persons aged 25–44 years, whereas among non-AI/AN persons, the largest percentage (35%) occurred among persons aged 45–64 years. Nearly 45% of AI/AN suicide decedents (compared with 18.7% of non-AI/AN suicide decedents) lived in nonmetropolitan areas. AI/AN suicide decedents had higher odds of dying by hanging, strangulation, or suffocation (aOR = 1.8) and lower odds of dying from a firearm injury (aOR = 0.7) compared with non-AI/AN decedents. AI/AN suicide decedents also had higher odds of dying in a natural area (e.g., field; aOR = 1.4) or supervised facility (e.g., prison; aOR = 2.0) compared with non-AI/AN suicide decedents.

**TABLE 1:** Selected demographic and descriptive characteristics of American Indian or Alaska Native and non–American Indian or Alaska Native suicide decedents — National Violent Death Reporting System, United States, 2015–2020

Characteristic	No. (%)*		Chi-square p-value^†^	aOR (95% CI)
	AI/AN (n = 3,397)	Non-AI/AN (n = 179,850)		
**Age group, yrs**				
10–14	83 (2.4)	1,996 (1.1)	<0.001	—**^§^**
15–19	368 (10.8)	8,591 (4.8)	<0.001	—**^§^**
20–24	491 (14.5)	14,440 (8.0)	<0.001	—**^§^**
25–44	1,593 (46.9)	58,662 (32.6)	<0.001	—**^§^**
45–64	701 (20.6)	62,941 (35.0)	<0.001	—**^§^**
≥65	161 (4.7)	33,220 (18.5)	<0.001	—**^§^**
**Sex**				
Male	2,553 (75.2)	140,690 (78.2)	<0.001	—**^§^**
Female	844 (24.8)	39,155 (21.8)	<0.001	—**^§^**
**Ethnicity**				
Hispanic or Latino	233 (6.9)	13,486 (7.5)	0.154	0.7 (0.6–0.8)^¶^
Non-Hispanic	3150 (93.1)	165,358 (92.5)	0.154	1.4 (1.2–1.6)^¶^
RUCA**				
Nonmetro	1,515 (44.8)	33,476 (18.7)	<0.001	3.7 (3.4–3.9)^¶^
Metro	1,864 (55.2)	145,345 (81.3)	<0.001	0.3 (0.3–0.3)^¶^
**Method**				
Firearm	1,261 (37.1)	88,893 (49.4)	<0.001	0.7 (0.6–0.7)^¶^
Hanging, strangulation, or suffocation	1,594 (46.9)	51,457 (28.6)	<0.001	1.8 (1.7–1.9)^¶^
Poisoning	312 (9.2)	23,309 (13.0)	<0.001	0.7 (0.7–0.8)^¶^
Motor vehicle	62 (1.8)	2,925 (1.6)	0.365	0.9 (0.7–1.2)
Sharp instrument	74 (2.2)	3,577 (2.0)	0.434	1.4 (1.1–1.7)^¶^
Fall	43 (1.3)	4,628 (2.6)	<0.001	0.5 (0.3–0.6)^¶^
Other (single method)	30 (0.9)	3,063 (1.7)	<0.001	0.5 (0.4–0.8)^¶^
**Location of injury**				
House or apartment	2,373 (69.9)	130,802 (72.7)	<0.001	0.9 (0.8–1.0)^¶^
Transport related^††^	272 (8.0)	19,004 (10.6)	<0.001	0.7 (0.6–0.8)^¶^
Natural area**^§§^**	236 (6.9)	8,368 (4.7)	<0.001	1.4 (1.2–1.6)^¶^
Supervised facility^¶¶^	99 (2.9)	2,512 (1.4)	<0.001	2.0 (1.7–2.5)^¶^
Hotel or motel	72 (2.1)	4,060 (2.3)	0.592	1.0 (0.8–1.3)
Abandoned building or industrial setting***	22 (0.6)	762 (0.4)	0.048	1.5 (1.0–2.3)
School (including college)	19 (0.6)	460 (0.3)	<0.001	1.1 (0.7–1.8)
Other	228 (6.7)	9,107 (5.1)	<0.001	1.3 (1.2–1.5)^¶^
**Other characteristic**				
Current or former military personnel	271 (8.7)	28,912 (17.1)	<0.001	0.7 (0.6–0.8)^¶^
Current experience of homelessness	106 (3.3)	2,416 (1.4)	<0.001	2.4 (2.0–3.0)^¶^

**Abbreviations:** AI/AN = American Indian or Alaska Native; aOR = adjusted odds ratio; RUCA = rural-urban commuting area.

* Denominator includes all suicide decedents.

**^†^** Pearson’s chi-square test p-value for difference between AI/AN and non-AI/AN populations.

**^§^** aORs measure the association between the decedent having the demographic or incident characteristic and the race of the decedent being AI/AN. Each aOR used non-AI/AN as the referent group and controlled for age group and sex. Therefore, aORs for age groups and sex are not presented.

^¶^ p<0.05 for aOR significance test.

** Zip code RUCA codes (2010) were used to determine whether a decedent lived in a nonmetropolitan versus a metropolitan area. Decedent residential zip codes were dichotomized as metropolitan (RUCA codes 1–3) and nonmetropolitan (RUCA codes 4–10). https://www.ers.usda.gov/data-products/rural-urban-commuting-area-codes/documentation/

^††^ Includes suicides that occurred in a motor vehicle, street, highway, parking lot or garage, public transport, railroad tracks, or bridge.

**^§§^** Includes suicides that occurred on a beach or in a river, field, or woods.

^¶¶^ Includes suicides that occurred in jail, prison, or supervised residential facility.

*** Includes suicides that occurred in industrial or construction sites or an abandoned house, building, or warehouse.

The circumstances of suicide were known for 86% of AI/AN and 89% of non-AI/AN decedents (Table 2). AI/AN decedents were more likely than were non-AI/AN decedents to disclose suicidal intent before death (aOR = 1.2) and to have had previous suicidal thoughts or plans (aOR = 1.1), but they were less likely to leave a note (aOR = 0.7). Nearly 55% of AI/AN suicide decedents experienced any relationship problems or losses before their death, compared with 42.2% of non-AI/AN decedents (aOR = 1.4). AI/AN decedents had increased odds of an additional nine of 14 relationship problems, including higher odds of intimate partner problems (aOR = 1.4), family relationship problems (aOR = 1.2), other relationship problems (aOR=1.4), interpersonal violence victimization (aOR = 2.7) and perpetration (aOR = 1.6) within the preceding month, suicide of a friend or family member (aOR = 1.6), and arguments or conflicts preceding death (aOR = 1.6). Conversely, AI/AN suicide decedents had decreased odds of physical health, job, and financial problems than did non-AI/AN decedents (aOR range = 0.6–0.8).

**TABLE 2:** Circumstances preceding suicide of American Indian or Alaska Native persons compared with non–American Indian or Alaska Native persons — National Violent Death Reporting System, United States, 2015–2020

Circumstance	No. (%)*		Chi-square p-value^†^	aOR (95% CI)^§^
	AI/AN (n = 3,397)	Non-AI/AN (n = 179,850)		
**Decedents with known circumstance** ^¶^	2,926 (86.1)	160,165 (89.1)	<0.001	0.8 (0.7–0.9)**
**Suicide event or history**				
Left a note	737 (25.2)	52,401 (32.7)	<0.001	0.7 (0.6–0.8)**
Disclosed suicidal intent	825 (28.2)	37,837 (23.6)	<0.001	1.2 (1.1–1.3)**
History of suicidal thoughts or plan	1,122 (38.3)	54,972 (34.3)	<0.001	1.1 (1.0–1.2)**
History of suicide attempts	634 (21.7)	31,608 (19.7)	0.009	1.0 (0.9–1.1)
**Relationship problem or loss**				
Any relationship problem or loss	1,607 (54.9)	67,542 (42.2)	<0.001	1.4 (1.3–1.6)**
Intimate partner problem	1,062 (36.3)	42,912 (26.8)	<0.001	1.4 (1.3–1.5)**
Family relationship problem	377 (12.9)	13,993 (8.7)	<0.001	1.2 (1.1–1.3)**
Other relationship problem (nonintimate)	121 (4.1)	3,467 (2.2)	<0.001	1.4 (1.2–1.7)**
Victim of interpersonal violence within previous mo	42 (1.4)	684 (0.4)	<0.001	2.7 (1.9–3.7)**
Perpetrator of interpersonal violence within previous mo	105 (3.6)	3,642 (2.3)	<0.001	1.6 (1.3–2.0)**
Suicide of friend or family member	123 (4.2)	3,787 (2.4)	<0.001	1.6 (1.3–1.9)**
Other death of friend or family member	180 (6.2)	10,122 (6.3)	0.711	1.1 (1.0–1.3)
Argument or conflict preceded death^††^	762 (26.0)	25,620 (16.0)	<0.001	1.6 (1.5–1.7)**
Injury occurred during argument	164 (21.5)	5,674 (22.1)	0.682	1.0 (0.9–1.2)
Injury occurred ≤24 hrs, but not during argument	502 (65.9)	15,721 (61.4)	0.012	1.1 (1.0–1.3)
Injury occurred >24 hrs after argument	57 (7.5)	2,715 (10.6)	0.006	0.7 (0.5–0.9)**
**Other life stressor**				
Any life stressor	1,640 (56.0)	94,851 (59.2)	0.001	1.0 (0.9–1.1)
Victim in custody	132 (4.5)	4,037 (2.5)	<0.001	1.7 (1.4–2.0)**
Released from institution within previous month^§§^	196 (6.7)	11,232 (7.0)	0.509	1.0 (0.9–1.2)
Jail, prison, or a detention facility	70 (35.7)	1,822 (16.2)	<0.001	2.5 (1.8–3.3)**
Hospital	55 (28.1)	4,559 (40.6)	<0.001	0.7 (0.5–1.0)
Psychiatric hospital or other psychiatric institution	36 (18.4)	3,589 (32.0)	<0.001	0.4 (0.3–0.6)**
Long-term residential health facility	2 (1.0)	126 (1.1)	1.000	1.6 (0.4–6.5)
Supervised residential facility related to alcohol or substance use treatment	18 (9.2)	622 (5.5)	0.028	1.5 (0.9–2.5)
Other^§§,¶¶^	15 (7.7)	514 (4.6)	0.042	1.6 (1.0–2.8)
Criminal legal problem	347 (11.9)	12,384 (7.7)	<0.001	1.6 (1.4–1.8)**
Civil legal problem	127 (4.3)	5,391 (3.4)	0.004	1.4 (1.1–1.6)**
Physical health problem	366 (12.5)	3,4291 (21.4)	<0.001	0.8 (0.7–0.9)**
Job problem***	179 (6.6)	15,092 (9.8)	<0.001	0.6 (0.6–0.8)**
Financial problem***	160 (5.9)	13,097 (8.5)	<0.001	0.8 (0.6–0.9)**
School problem^†††^	53 (17.5)	1,586 (21.6)	<0.001	0.8 (0.6–1.1)
Eviction or loss of home	93 (3.2)	5,638 (3.5)	<0.001	1.0 (0.8–1.3)
**Crisis within previous 2 wks or anticipated in upcoming 2 wks**	930 (31.8)	475,96 (29.7)	0.015	1.1 (1.0–1.2)
Crisis related to mental health^§§§^	21 (2.3)	2,679 (5.6)	<0.001	0.4 (0.2–0.6)**
Crisis related to alcohol problem^§§§^	74 (8.0)	2,718 (5.7)	0.004	1.6 (1.3–2.0)**
Crisis related to substance use^§§§^	33 (3.5)	1,619 (3.4)	0.807	1.0 (0.7–1.4)
Crisis related to intimate partner problem^§§§^	417 (44.8)	18,278 (38.4)	<0.001	1.2 (1.0–1.3)**
Crisis related to family relationship problem^§§§^	68 (7.3)	3,589 (7.5)	0.794	0.7 (0.5–0.9)**
Crisis related to other relationship problem^§§§^	19 (2.0)	799 (1.7)	0.393	0.9 (0.6–1.5)
Crisis related to criminal legal problem^§§§^	140 (15.1)	5,536 (11.6)	0.001	1.4 (1.1–1.6)**
Crisis related to civil legal problem^§§§^	31 (3.3)	1,628 (3.4)	0.885	1.0 (0.7–1.5)
Crisis related to physical health problem^§§§^	85 (9.1)	7,067 (14.8)	<0.001	1.0 (0.8–1.2)
Crisis related to job problem***^,§§§^	34 (4.0)	3,509 (7.7)	<0.001	0.5 (0.3–0.7)**
Crisis related to financial problem***^,§§§^	16 (1.9)	2,069 (4.5)	<0.001	0.5 (0.3–0.8)**
Crisis related to school problem^†††,§§§^	12 (12.8)	404 (17.7)	0.218	0.7 (0.4–1.3)
Crisis related to eviction or loss of home^§§§^	25 (2.7)	2,290 (4.8)	0.003	0.6 (0.4–1.0)**
Crisis related to recent suicide of friend or family^§§§^	39 (4.2)	442 (0.9)	<0.001	3.8 (2.7–5.3)**
Crisis related to other death of friend or family^§§§^	38 (4.1)	1,710 (3.6)	0.424	1.3 (0.9–1.8)
**Mental health or substance use**				
Any current substance use problem	1,340 (45.8)	47,285 (29.5)	<0.001	2.0 (1.9–2.2)**
Alcohol problem	918 (31.4)	29,109 (18.2)	<0.001	2.3 (2.1–2.5)**
Other substance use problem	778 (26.6)	27,403 (17.1)	<0.001	1.6 (1.5–1.7)**
Reported alcohol use in hrs preceding death	902 (30.8)	31,185 (19.5)	<0.001	1.9 (1.7–2.0)**
Any current diagnosed mental health problem	1,215 (41.5)	78,744 (49.2)	<0.001	0.7 (0.7–0.8)**
Depression or dysthymia	859 (29.4)	58,580 (36.6)	<0.001	0.7 (0.7–0.8)**
Bipolar disorder	146 (5.0)	11,776 (7.4)	<0.001	0.6 (0.5–0.8)**
Schizophrenia	100 (3.4)	4,714 (2.9)	0.133	1.1 (0.9–1.4)
Anxiety disorder	219 (7.5)	15,810 (9.9)	<0.001	0.7 (0.6–0.8)**
Posttraumatic stress disorder	92 (3.1)	4,235 (2.6)	0.095	1.2 (1.0–1.5)
Attention deficit hyperactivity disorder	37 (1.3)	2,161 (1.3)	0.694	0.5 (0.4–0.7)**
Current depressed mood (not diagnosis)	986 (33.7)	55,385 (34.6)	0.320	1.0 (0.9–1.1)
**Mental health or substance use treatment**				
Current mental health or substance use treatment	569 (19.4)	41,894 (26.2)	<0.001	0.6 (0.6–0.7)**
History of mental health or substance use treatment	862 (29.5)	56,260 (35.1)	<0.001	0.7 (0.7–0.8)**

**Abbreviations:** AI/AN = American Indian or Alaska Native; aOR = adjusted odds ratio.

* Denominator includes all suicide decedents.

^†^ Pearson’s chi-square test result for difference between AI/AN and non-AI/AN populations; Fisher's exact test when one or more of the cell counts in a 2×2 table is <5.

^§^ aORs measure the association between the decedent having the precipitating circumstance present and the race of the decedent being AI/AN. Each aOR used Non-AI/AN as the referent group and controlled for age group and sex.

^¶^ Denominator includes only suicides with one or more precipitating circumstance, unless otherwise noted. Sum of percentages in columns might exceed 100% because a suicide could have more than one precipitating circumstance.

** p<0.05 for aOR significance test.

^††^ Denominator includes only those suicides in which argument or conflict preceded death.

^§§^ Denominator includes only those decedents released from an institution within the previous month.

^¶¶^ Supervised residential facilities not related to alcohol or substance use treatment, and other or unknown type of institution.

*** Denominator includes only decedents aged ≥18 years with at least one known circumstance.

^†††^ Denominator includes only decedents aged 10–17 years with at least one known circumstance.

^§§§^ Denominator includes only those suicide decedents with any crisis within the past or upcoming 2 weeks.

Approximately one third of AI/AN (31.8%) and non-AI/AN suicide decedents (29.7%) had experienced a crisis within the preceding 2 weeks or anticipated a crisis in the upcoming 2 weeks; AI/AN decedents had higher odds of having experienced crises involving intimate partners and recent suicide of friends or family members as well as crises involving criminal legal problems than did non-AI/AN decedents (aOR range = 1.2–3.8). In addition, AI/AN decedents had higher odds of six of seven alcohol or substance use problems including any current substance use problem (aOR = 2.0), a current alcohol (aOR = 2.3) or other substance use problem (aOR = 1.6), reported alcohol use hours before death (aOR = 1.9), and crises involving alcohol (aOR = 1.6). Among persons released from an institution within the month preceding death (196), 9.2% of AI/AN decedents had been in residential substance use treatment, compared with 5.5% of non-AI/AN decedents. The prevalences of known mental health diagnoses (41.5%; [aOR = 0.7]) and history of mental health or substance use treatment (29.5%; [aOR = 0.7]) were lower among AI/AN decedents than among non-AI/AN decedents (49.2% and 35.1%, respectively).

Toxicology testing was performed for 66.6% of AI/AN suicide decedents and 61.1% of non-AI/AN decedents (Table 3). Overall, AI/AN decedents had higher odds than did non-AI/AN decedents of receiving a positive test result for at least one substance (aOR = 1.2), blood alcohol concentration of ≥0.08 g/dL (aOR = 2.3), amphetamines (aOR = 1.5), and marijuana (aOR = 1.5). Conversely, AI/AN decedents had lower odds than did non-AI/AN decedents of receiving a positive test result for opioids (aOR = 0.5), benzodiazepines (aOR = 0.4), cocaine (aOR = 0.5), antidepressants (aOR = 0.6), antipsychotics (aOR = 0.7), and barbiturates (aOR = 0.3).

**TABLE 3:** Toxicology results of American Indian or Alaska Native suicide decedents compared with non–American Indian or Alaska Native suicide decedents — National Violent Death Reporting System, United States, 2015–2020

Toxicology result	No. (%)		Chi-square p-value^†^	aOR (95% CI)^§^
	AI/AN (n = 3,397)	Non-AI/AN (n = 179,850)		
Any toxicology testing*	2,262 (66.6)	109,806 (61.1)	<0.001	1.2 (1.1–1.3)^¶^
Positive result for at least one substance**	1,774 (78.4)	84,152 (76.6)	0.046	1.2 (1.1–1.3)^¶^
**Blood alcohol concentration^††^**				
Tested	2,103 (61.9)	93,124 (51.8)	<0.001	1.4 (1.3–1.5)^¶^
Positive result**^§§^**	1,023 (48.6)	37,354 (40.1)	<0.001	1.5 (1.4–1.7)^¶^
Alcohol <0.08 g/dL	169 (16.5)	10,667 (28.6)	<0.001	0.5 (0.4–0.6)^¶^
Alcohol ≥0.08 g/dL	821 (80.3)	24,019 (64.3)	<0.001	2.3 (2.0–2.7)^¶^
Alcohol positive, level unknown	33 (3.2)	2,668 (7.1)	<0.001	0.5 (0.3–0.6)^¶^
**Opioids**				
Tested	1,842 (54.2)	76,672 (42.6)	<0.001	1.5 (1.4–1.6)^¶^
Positive result**^§§^**	228 (12.4)	18,242 (23.8)	<0.001	0.5 (0.5–0.6)^¶^
**Benzodiazepines**				
Tested	1,713 (50.4)	71,192 (39.6)	<0.001	1.5 (1.4–1.6)^¶^
Positive result**^§§^**	190 (11.1)	18,511 (26.0)	<0.001	0.4 (0.3–0.5)^¶^
**Cocaine**				
Tested	1,796 (52.9)	72,121 (40.1)	<0.001	1.6 (1.5–1.7)^¶^
Positive result**^§§^**	64 (3.6)	4,940 (6.8)	<0.001	0.5 (0.4–0.6)^¶^
**Amphetamines**				
Tested	1,841 (54.2)	70,483 (39.2)	<0.001	1.7 (1.6–1.8)^¶^
Positive result**^§§^**	381 (20.7)	9,523 (13.5)	<0.001	1.5 (1.4–1.7)^¶^
**Marijuana**				
Tested	1,363 (40.1)	62,684 (34.9)	<0.001	1.2 (1.1–1.2)^¶^
Positive result**^§§^**	491 (36.0)	15,102 (24.1)	<0.001	1.5 (1.3–1.7)^¶^
**Antidepressants**				
Tested	804 (23.7)	48,972 (27.2)	<0.001	0.8 (0.7–0.9)^¶^
Positive result**^§§^**	203 (25.2)	18,294 (37.4)	<0.001	0.6 (0.5–0.7)^¶^
**Antipsychotics**				
Tested	690 (20.3)	38,001 (21.1)	0.248	0.9 (0.8–1.0)
Positive result**^§§^**	54 (7.8)	4,336 (11.4)	0.003	0.7 (0.5–0.9)^¶^
**Barbiturates**				
Tested	1,651 (48.6)	59,040 (32.8)	<0.001	1.8 (1.7–2.0)^¶^
Positive result**^§§^**	11 (0.7)	1,441 (2.4)	<0.001	0.3 (0.2–0.6)^¶^
**Carbon monoxide**				
Tested	133 (3.9)	10,333 (5.7)	<0.001	0.7 (0.6–0.8)^¶^
Positive result**^§§^**	41 (30.8)	3,492 (33.8)	0.472	1.0 (0.7–1.5)
**Anticonvulsants**				
Tested	547 (16.1)	38,439 (21.4)	<0.001	0.7 (0.6–0.8)^¶^
Positive result**^§§^**	77 (14.1)	6,487 (16.9)	0.082	0.8 (0.6–1.1)
**Muscle relaxants**				
Tested	492 (14.5)	39,311 (21.9)	<0.001	0.6 (0.5–0.6)^¶^
Positive result**^§§^**	34 (6.9)	2,575 (6.6)	0.748	1.1 (0.8–1.6)

**Abbreviations:** AI/AN = American Indian or Alaska Native; aOR = adjusted odds ratio.

* Denominator includes all suicide decedents.

**^†^** Pearson’s chi-square test result for difference between AI/AN and non-AI/AN populations.

^§^ aORs measure the association between the decedent receiving a positive test result for the substance and the race of the decedent being AI/AN. The denominator was the number of decedents who were tested for each substance. Each aOR used non-AI/AN as the referent group and controlled for age group and sex.

**^¶^** p<0.05 for aOR significance test.

** Denominator is decedents with any toxicology testing.

**^††^** Blood alcohol concentration of ≥0.08 g/dL is higher than the legal limit in all states and the District of Columbia and is used as the standard for intoxication.

**^§§^** Denominator for each positive result group is the number tested for the substance in that group.

## Discussion

Analyses of characteristics of and circumstances preceding suicide among AI/AN and non-AI/AN persons in participating NVDRS jurisdictions during 2015–2020 identified many differences, including higher odds of relationship and substance use problems and lower odds of physical, job, and financial problems; known mental health conditions; and any history of mental health or substance use treatment among AI/AN decedents compared with non-AI/AN decedents. Although direct comparison of circumstances between studies is not possible, these findings suggest a similar pattern observed in a previous analysis of suicide in 18 states among non-Hispanic AI/AN persons compared with non-Hispanic White populations, during 2003–2014 (*2*). Those findings also indicated higher odds of relationship and alcohol problems and reduced odds of known mental health problems, current or past mental health or substance use treatment, and physical, job, or financial problems. Toxicology results from the earlier study also followed the same pattern as those observed in the current study, including higher odds of positive alcohol, amphetamine, and marijuana toxicology results among AI/AN decedents, and reduced odds of positive opioid and antidepressant test results, compared with non-AI/AN decedents.

The current study found higher odds of suicide among AI/AN persons across a range of relationship problems related to intimate partners, family, other relationships, interpersonal violence victimization and perpetration, and death of friends or family members by suicide. Similarly, more alcohol and other substance use circumstances, including those of an acute and more chronic nature, were observed in this study, as were criminal problems, although the nature of these problems was unknown. According to previous NVDRS reports, approximately one half of persons who die by suicide do not have a known mental health condition (*4*). This study found that only 41.5% of AI/AN suicide decedents had a known mental health condition. This might be the result of less available or accessible mental health services, especially in rural areas, and therefore fewer diagnoses. Post-hoc analyses controlling for metropolitan status did not change these results, suggesting possible contribution of other factors.

Suicide prevention efforts among AI/AN populations must consider the context and consequences of current inequities as well as historical trauma, including intergenerational transmission, that continue to affect AI/AN persons, families, and communities today (*8*). Suicide is a complex problem with multiple contributing circumstances that affect different communities differently. A comprehensive public health approach to suicide prevention (*3*), with attention to strategies that aim to reduce health inequities among AI/AN persons, is needed. These strategies might include strengthening access to and delivery of culturally relevant care, including telehealth for mental health concerns and well-being, increasing training and hiring of AI/AN providers, promoting community engagement and cultural traditions, increasing coping and problem-solving skills (e.g., American Indian Life Skills Training),^§^ increasing training to recognize and respond to suicide risk, making postvention programs (activities that reduce risk and promote healing after a suicide death) more available to AI/AN survivors of suicide loss (*3*), and promoting the 988 Suicide and Crisis Lifeline (persons who are thinking about suicide or who know someone who is thinking about suicide, should call 988).^¶^

The findings in this report are subject to at least four limitations. First, participation in NVDRS states increased during the analysis period; therefore, not all jurisdictions contributed data equally during all years. Second, deaths among AI/AN persons are prone to racial and ethnic misclassification, leading to potential underestimation of AI/AN suicides (*9*). However, the analysis included any decedent with noted AI/AN ancestry, including multiracial AI/AN, irrespective of Hispanic ethnicity, allowing for a more inclusive understanding of AI/AN suicide characteristics and circumstances. Third, NVDRS does not yet include tribal affiliation, and results might vary by tribe. Finally, circumstance data in NVDRS rely upon reporting by next-of-kin and other informants who knew the decedent, and their knowledge and willingness to share information about the decedent and circumstances preceding suicide. This might overestimate or underestimate this information.

Prevention of suicide is possible (*3*). Identification of new evidence-based programs, evaluation of existing AI/AN programs, and tailoring of other effective programs to prevent suicide among AI/AN persons is needed. Programs can benefit from holistic indigenous evaluation, which takes into consideration AI/AN cultural values and practices, such as storytelling (*10*). Addressing AI/AN-specific risk and promoting the many protective factors among AI/AN persons can save lives.

**Summary**

**What is already known about this topic?**

Suicide is preventable. It disproportionately affects American Indian or Alaska Native (AI/AN) persons. Previous studies have examined suicide characteristics and circumstances among non-Hispanic AI/AN only in a limited number of states.

**What is added by this report?**

Comparison of 2015–2020 suicides among all AI/AN and non-AI/AN decedents in 49 states, Puerto Rico, and the District of Columbia found that AI/AN suicide decedents had higher adjusted odds of a range of relationship and alcohol or other substance use problems, and reduced odds of known mental health conditions and treatment than did non-AI/AN suicide decedents.

**What are the implications for public health practice?**

Culturally relevant comprehensive public health approaches to suicide prevention are needed to address systemic and long-standing inequities among AI/AN persons.

## Footnotes

* Florida is not included because pilot data were collected only during the study period.

† 45 C.F.R. part 46, 21 C.F.R. part 56; 42 U.S.C. Sect. 241(d); 5 U.S.C. Sect. 552a; 44 U.S.C. Sect. 3501 et seq.

§
https://www.sprc.org/resources-programs/american-indian-life-skills-developmentzuni-life-skills-development

¶
www.988lifeline.org
